# Overexpression and Small Molecule-Triggered Downregulation of CIP2A in Lung Cancer

**DOI:** 10.1371/journal.pone.0020159

**Published:** 2011-05-31

**Authors:** Liang Ma, Zhe-Sheng Wen, Zi Liu, Zheng Hu, Jun Ma, Xiao-Qin Chen, Yong-Qiang Liu, Jian-Xin Pu, Wei-Lie Xiao, Han-Dong Sun, Guang-Biao Zhou

**Affiliations:** 1 Division of Molecular Carcinogenesis and Targeted Therapy for Cancer, State Key Laboratory of Biomembrane and Membrane Biotechnology, Institute of Zoology, Chinese Academy of Sciences, Beijing, China; 2 School of Life Sciences, University of Science and Technology of China, Hefei, China; 3 The Cancer Hospital, Sun Yat-Sen University, Guangzhou, China; 4 Guangzhou Institute of Biomedicine and Health, Chinese Academy of Sciences, Guangzhou, China; 5 State Key Laboratory of Phytochemistry and Plant Resources in West China, Kunming Institute of Botany, Chinese Academy of Sciences, Kunming, China; Univesity of Texas Southwestern Medical Center at Dallas, United States of America

## Abstract

**Background:**

Lung cancer is the leading cause of cancer deaths worldwide, with a five-year overall survival rate of only 15%. Cancerous inhibitor of PP2A (CIP2A) is a human oncoprotein inhibiting PP2A in many human malignancies. However, whether CIP2A can be a new drug target for lung cancer is largely unclear.

**Methodology/Principal Findings:**

Normal and malignant lung tissues were derived from 60 lung cancer patients from southern China. RT-PCR, Western blotting and immunohistochemistry were used to evaluate the expression of CIP2A. We found that among the 60 patients, CIP2A was undetectable or very low in paratumor normal tissues, but was dramatically elevated in tumor samples in 38 (63.3%) patients. CIP2A overexpression was associated with cigarette smoking. Silencing CIP2A by siRNA inhibited the proliferation and clonogenic activity of lung cancer cells. Intriguingly, we found a natural compound, rabdocoetsin B which is extracted from a Traditional Chinese Medicinal herb Rabdosia coetsa, could induce down-regulation of CIP2A and inactivation of Akt pathway, and inhibit proliferation and induce apoptosis in a variety of lung cancer cells.

**Conclusions/Significance:**

Our findings strongly indicate that CIP2A could be an effective target for lung cancer drug development, and the therapeutic potentials of CIP2A-targeting agents warrant further investigation.

## Introduction

Nearly 1.5 million people were diagnosed with lung cancer and 1.4 million people were estimated to die from it in 2007 [1]. The two major forms of lung cancer are non–small cell lung cancer (NSCLC) and small cell lung cancer (SCLC). About 85% of lung cancers are NSCLC, which can be divided into three major histological subtypes: squamous cell carcinoma, adenocarcinoma, and large-cell carcinoma. SCLC represents about 15% of lung cancers [2]. Smoking causes all types of lung cancer but is most strongly linked with SCLC and squamous-cell carcinoma; adenocarcinoma is the most common type in patients who have never smoked [2]. Current treatment is determined by the histologic type of lung cancer and stage at diagnosis, including surgery, platinum doublet therapy, radiation therapy and targeted therapy. Unfortunately, the prognosis of lung cancer is poor with a only 15% of five-year overall survival rate for all stages combined [1]. Therefore, there is an urgent need to identify more effective molecular targets and new targeted therapies for lung cancer.

Cancerous Inhibitor of PP2A (CIP2A) is a human oncoprotein that stabilizes c-Myc by inhibiting protein phosphatase 2A (PP2A)-mediated dephosphorylation of MYC at serine 62 [3]. In addition to inhibiting the degradation of c-Myc, CIP2A appears to be regulated in a positive feedback loop with c-Myc by promoting each other's expression [4]. CIP2A is over-expressed in human neck and head carcinomas, colon, breast, and gastric cancer, and is inversely correlated with disease outcome in gastric cancer [3]–[7]. However, whether CIP2A could be a new drug target for cancers is not fully investigated, and the anti-tumor activity of CIP2A-targeting agents remains largely unknown. We studied the expression of CIP2A in lung cancer and screened for lead compounds that could target CIP2A [8]. Here we show that CIP2A is markedly upregulated in lung cancer tumors compared to patient-matched adjacent normal lung tissues, and report that a natural compound which triggers downregulation of CIP2A exhibits significant antitumor activity in NSCLC cell lines.

## Results

### CIP2A is over-expressed in lung cancer and is associated with cigarette smoking

**Figure 1:**
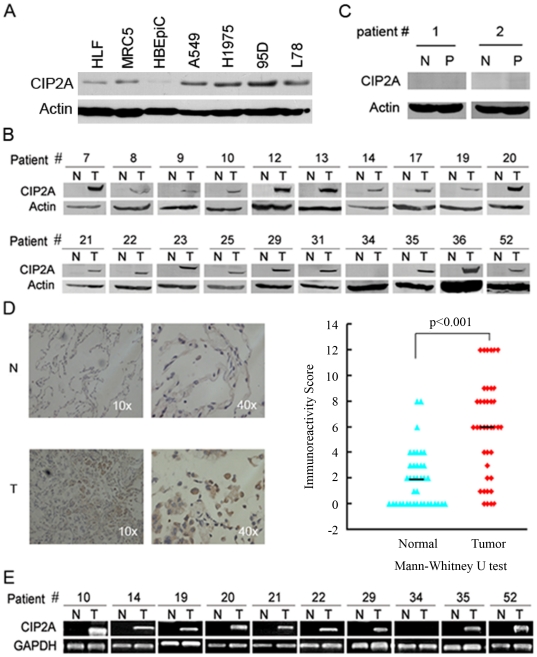
CIP2A is over-expressed in human lung cancer. (A): Western blot analysis of CIP2A in human lung cancer cells (A549, H1975, 95D and L78), embryonic lung fibroblast cells (HLF and MRC-5), and normal human bronchial epithelial cells (HBEpiC). (B): Western blot analyses of CIP2A protein in primary lung tumors (T) and adjacent normal lung tissues (N). β-Actin is used as a loading control. Representative results are shown and numbers are referred to individual patients. (C): Western blot analyses of CIP2A protein in inflammatory pseudotumor (P) and adjacent normal lung tissues (N). β-Actin is used as a loading control. (D): Representative images (left panel) and score (right panel) of immunohistochemistry staining for CIP2A expression in primary lung tumors (T) and adjacent normal lung tissues (N). (E): RT-PCR analyses of *CIP2A* mRNA in primary lung tumors (T) and adjacent normal lung tissues (N). *GAPDH* is employed as a loading control. Representative results are shown and numbers are referred to individual patients.

We tested the expression of CIP2A at protein level in nonmalignant and malignant cells, and found that CIP2A was highly expressed in lung cancer cell lines (A549, H1975, 95D and L78) compared to normal human embryonic lung fibroblasts (HLF and MRC5) and normal human bronchial epithelial cells (HBEpiC) (Figure 1A). We then analyzed CIP2A in 60 lung cancer samples from patients came from southern China whose baseline characteristics were listed in Table 1. We showed that CIP2A was over-expressed in 38 (63.3%) tumor specimens assayed by western blotting (Figure 1B). However, in the 60 patient-matched adjacent normal lung tissues, CIP2A was undetectable in 57 (95%) samples, and was weakly expressed in 3 (5%) specimens where its expression was much lower than that in tumor samples of the same patients. In samples from 2 patients with inflammatory pseudotumor, CIP2A was not detected in both the pseudotumor and adjacent lung tissues (Figure 1C). Immunohistochemistry assay confirmed that CIP2A was dramatically elevated with a higher immunoreactivity score in tumor samples in 26 out of 39 patients (66.7%) tested (Figure 1D). At mRNA level, *CIP2A* was also over-expressed in lung tumor tissues compared to normal lung tissues in 39 of 58 (67.2%) patients tested (Figure 1E). Taken together, CIP2A is dramatically elevated in lung cancer tumor samples compared to paired normal lung tissues.

**Table 1:** Baseline demographic characteristics of patients underwent western blot analysis of CIP2A.

Characteristics	Cases, n	CIP2A-high, n (%)	P* value
**Gender**			
male	40	30 (75)	0.008
female	20	8 (40)	
**Age**			
<65	44	25 (56.8)	0.082
> = 65	16	13 (81.2)	
**Smoking**			
smoker	36	28 (77.8)	0.004
non-smoker	24	10 (41.7)	
**Histology**			
adenocarcinoma	35	17 (48.6)	0.003**
squamous-cell carcinoma	19	17 (89.5)	
adenosquamouscarcinoma	5	3 (60)	
small cell lung cancer	1	1 (100)	
**TNM stage**			
IA	3	2 (66.7)	0.639
IIB	22	11 (50)	
IIA	2	1 (50)	
IIB	6	5 (83.3)	
IIIA	19	12 (63.2)	
IIIB	1	1 (100)	
IV	3	3 (100)	
NA	4	3 (75)	

*Chi-Square Test;

**Adenocarcinoma versus Squamous-cell carcinoma; NA, unknown.

### CIP2A overexpression is associated with cigarette smoking

We analyzed the correlation between CIP2A overexpression and some clinicopathologic variables. The data demonstrated that CIP2A overexpression in lung cancer was significantly correlated with histologic type of squamous cell carcinoma (p = 0.003) and male gender (p = 0.008) (Table 1) that were shown to strongly link with cigarette smoking [2], [9]. Moreover, our data clearly showed that the high expression of CIP2A was associated with smoker patients (p = 0.004). No significant difference in the CIP2A status was observed according to the pathological stage (p = 0.639) and age (0.082) (Table 1). In an attempt to clarify the most significant factors related to CIP2A overexpression, the multivariate analyses were performed, and the multivariate logistic regression analysis (Table 2) demonstrated that cigarette smoking was the only significant variable associated with CIP2A overexpression in lung cancer (p = 0.008).

**Figure 2:**
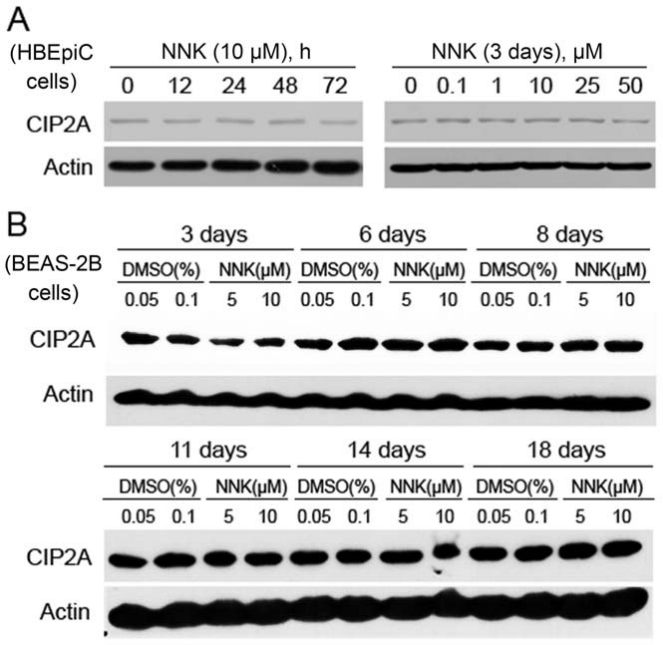
Effects of NNK on CIP2A expression. (A): HBEpiC cells were treated with NNK at various concentrations for indicated time points, and western blot was performed to analyze CIP2A expression. (B): BEAS-2B cells were treated with NNK at indicated concentrations for indicated time points, and western blot was conducted to analyze CIP2A expression. 0.05% and 0.1% DMSO were used as a solvent control corresponding to 5 µΜ and 10 µΜ NNK, respectively.

The nitrosamine 4-(methylnitro-samino)-1-(3-pyridyl)-1-butanone, or the nicotine-derived nitrosamine ketone (NNK), is a key ingredient of tobacco smoke carcinogen which systemically induces tumors of the lung in rats, mice, and hamsters and also plays a major role in lung carcinogenesis [10], [11]. We then investigated whether NNK could directly induce upregulation of CIP2A or not. To do this, HBEpiC (Figure 2A) and BEAS-2B (Figure 2B) bronchial epithelial cells were exposed to NNK at indicated concentration for indicated time points, lysed, and western blot was performed to analyze the expression of CIP2A. The results showed that treatment with NNK at 0.1 to 10 µM for up to 18 days could not perturb CIP2A expression (Figure 2, A and B). In this study, we did not test the long-term effect of NNK on CIP2A expression *in vitro* or *in vivo*.

**Table 2:** Multivariate logistic analyses of the association between CIP2A high expression and clinic characteristics.

Variable	Odds ratio	95.0% Confidence Interval	P value
**Smoking**	5.330	1.537–18.487	0.008
**Histology**	2.186	0.865–5.524	0.098
**TNM stage**	1.349	0.958–1.899	0.087

### CIP2A is required for lung cancer cells growth and transformation

**Figure 3:**
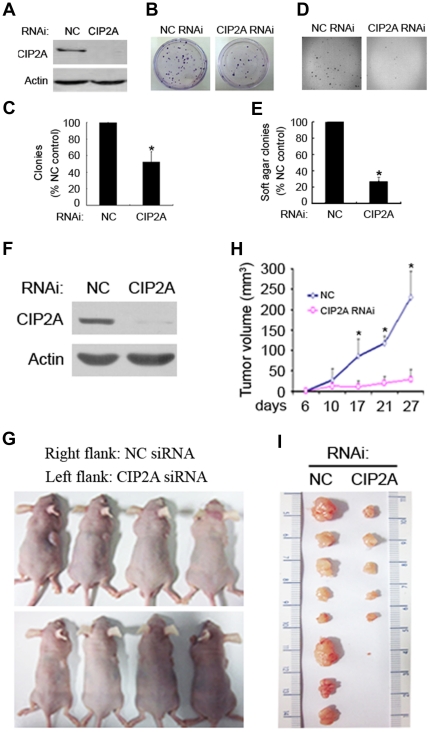
CIP2A is required for lung cancer cells' growth and transformation. (A): Western blot analysis of CIP2A protein expression in A549 cells 72 h after transfection with negative control (NC) or CIP2A-specific siRNA. (B and C): Flat plate clone formation assay for the clonogenic activity of A549 cells 72 h after transfection with NC or CIP2A-specific siRNA. (B): representative light miscroscopy images. (C): Quantitation of foci counting. Shown is mean+SD of four independent experiments. (D and E): Soft-agar colony formation assay for A549 cells transfected with NC or CIP2A-specific siRNA. (D): representative light miscroscopy images. (E): Quantitation of foci counting. (F): Western blot analysis of CIP2A protein in A549 cells transfected with NC or CIP2A-specific siRNA for 72 h. (G): Nude mice injected subcutaneously with A549 cells transfected with NC or CIP2A-specific siRNA. (H): The tumor growth curve for the experiment shown in (G). Shown is mean+SD of the mean tumor volumes. (I) Image of xenograft tumors obtained from mice shown in (G). * p<0.01, Student's t test.

CIP2A specific siRNA was employed to evaluate its roles in lung cancer pathogenesis, and the results showed that as compared to negative control (NC), CIP2A silencing (Figure 3A) led to inhibition of clonogenic activity of A549 cells, detected by foci formation (Figure 3, B and C) and soft agar colony formation (Figure 3, D and E) assays [3]. These phenomena were confirmed by results of CIP2A silencing in L78 cells (Figure S1, A through E). Next, A549 cells were transfected with NC or CIP2A-specific siRNA (Figure 3F), and injected subcutaneously into the right and left flanks of 8 nude mice respectively, and tumor volumes were estimated every two days [12]. Interestingly, CIP2A-specific siRNA significantly inhibited tumor growth as compared to NC-siRNA (Figure 3, G through I). Together, these data indicate that CIP2A is essential to lung cancer proliferation and tumorigenesis, and could be an effective therapeutic target.

### Rabdocoetsin B is a CIP2A-targeting natural compound

**Figure 4:**
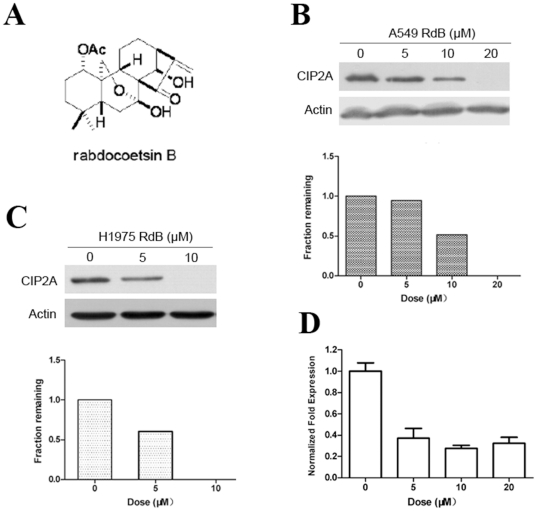
Rabdocoetsin B induces down-regulation of CIP2A. (A): Structure of rabdocoetsin B. (B): A549 cells were treated with rabdocoetsin B (RdB) at various concentrations for 48 h. Western blots were used to detect the expression of CIP2A protein (upper panel) and CIP2A protein expression were quantified and normalized against β-actin expression (lower panel) (C): H1975 cells were treated with rabdocoetsin B at various concentrations for 24 h. Western blots were used to detect the expression of CIP2A protein (upper panel) and CIP2A protein expression were quantified and normalized against β-actin expression (lower panel) (D): A549 cells were treated with rabdocoetsin B at various concentrations for 48 h, and the mRNA expression of *CIP2A* was analyzed using real-time RT-PCR.

Our central goal was to identify new drug targets and provide lead compounds for drug development. We screened for CIP2A-targeting small molecules by analyzing their effects on CIP2A expression, and identified a natural compound extracted from a Chinese medicinal herbal Rabdosia coetsa, rabdocoetsin B [13], [14] (Figure 4A), could downregulate CIP2A at protein level at 5 to 20 µM in A549 cells (Figure 4B). In H1975 cells, Rabdocoetsin B also triggered downregulation of CIP2A at 5 to 10 µM (Figure 4C). We analyzed the mechanism of CIP2A down-regulation caused by Rabdocoetsin B, and found that rabdocoetsin B significantly inhibited the transcription of CIP2A in a dose-dependent manner (Figure 4D) assessed by real-time RT-PCR.

### Rabdocoetsin B inhibits CIP2A-modulated phosphorylated Akt

**Figure 5:**
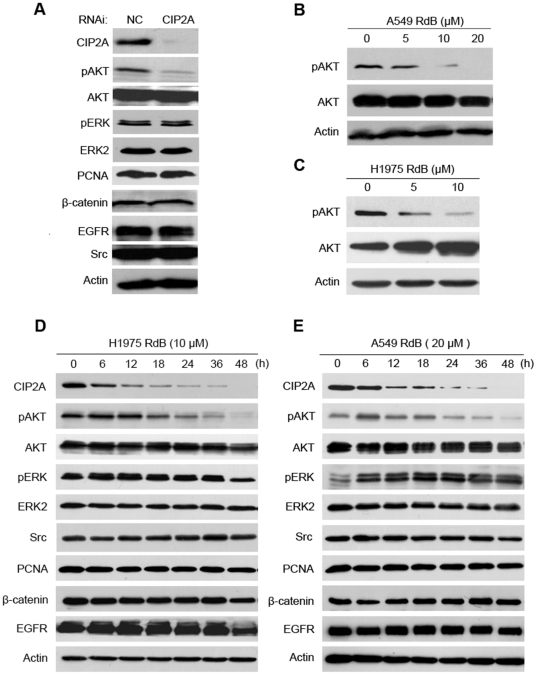
Downregulation of CIP2A leads to decrease of phosphorylated Akt in lung cancer cells. (A): A549 cells were transfected with NC or CIP2A-specific siRNA for 72 h, and the expression of indicated proteins were detected using Western blots. (B and C): A549 (B) and H1975 cells (C) were treated with rabdocoetsin B (RdB) at indicated concentrations for 48 and 24 h, respectively, and Western blots were performed to analyze the expression of proteins indicated. (D and E): H1975 (D) and A549 (E) cells were treated with rabdocoetsin B (RdB) for indicated time points, and Western blot analysis was carried out with antibodies specific for the indicated proteins. β-actin is used as a loading control.

In hepatocellular carcinoma cells, CIP2A up-regulates phospho-Akt (pAkt) and decreases Akt-related PP2A activity, whereas silencing CIP2A re-activates PP2A [15]. Since Akt is constitutively active in lung cancer cells and promotes cellular survival and resistance to chemotherapy and radiation [16], [17], we examined the effect of CIP2A on pAkt in lung cancer. We showed that CIP2A silencing by specific siRNA resulted in down-regulation of pAkt but not pERK, PCNA, β-catenin, EGFR or Src (Figure 5A). We then investigated whether rabdocoetsin B could modulate the expression of pAkt, and found that treatment with rabdocoetsin B at 5 to 10 µM also down-regulated pAkt in A549 (Figure 5B) and H1975 (Figure 5C) cells. We further showed that in H1975 cells upon rabdocoetsin B at 10 µM, CIP2A was markedly downregulated in 6 to 12 h and became undetectable in 48 h (Figure 5D), while pAKT was decreased in 18 h (Figure 5D). These results were confirmed in A549 cells treated with rabdocoetsin B (Figure 5E), indicating that this compound can inhibit the CIP2A-Akt pathway in lung cancer.

### Effects of Rabdocoetsin B on lung cancer cells

**Figure 6:**
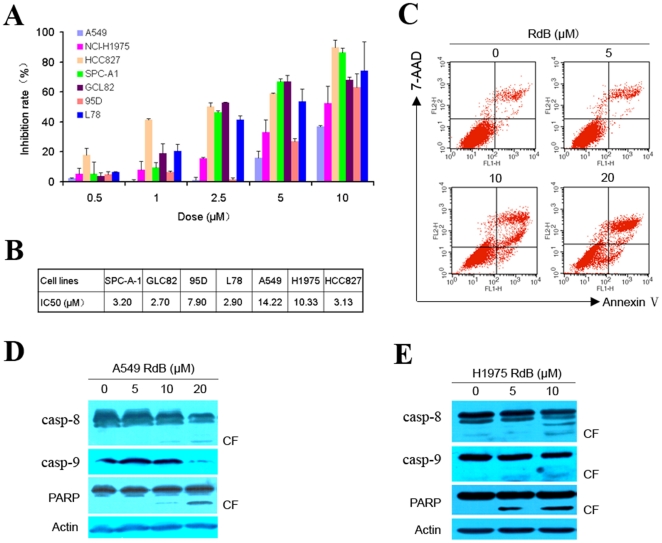
Rabdocoetsin B exhibits anti-tumor activity in lung cancer cells. (A): Lung cancer cells were treated with increasing concentrations of Rabdocoetsin B (RdB) (from 1 µM to 10 µM) and cell viability was measured 48 h by the MTT assay. (B): the IC50 of cells treated with rabdocoetsin B. (C): Rabdocoetsin B induces apoptosis of A549 cells, assayed by flow cytometry. (D and E): Western blots were performed to detect the expression of apoptosis regulators in A549 (D) and H1975 cells (E).

We evaluated the effects of rabdocoetsin B on lung cancer cells expressing wide-type (WT) or mutant EGFR. We showed that rabdocoetsin B exhibited significant cytotoxic effects on A549, NCI-H1975, HCC827, SPC-A-1, GLC-82, L78 and 95D lung cancer cell lines (Figure 6, A and B). Rabdocoetsin B induced apoptosis of A549 cells (Figure 6C) with activation of casp-8 and casp-9 and cleavage of PARP (Figure 6D). Rabdocoetsin B also caused activation of casp-8 and casp-9 and cleavage of PARP in NCI-H1975 cells (Figure 6E). These results indicate that rabdocoetsin B inhibits proliferation and induces apoptosis of lung cancer cells via activating the endogenous and exogenous apoptosis pathways.

## Discussion

CIP2A is an auto-antigen [7] that is over-expressed in human neoplasms [3]–[7], [18]–[21]. Peng [22] showed that in American-based patients, CIP2A is increased in 61 of the 72 (84.7%) lung cancer tissue specimens, which is significantly higher than in normal lung tissues (14.3%, 9/63). Recently, Dong et al [23] reported that in 29 patients from Northern China, *CIP2A* is over-expressed at mRNA level in 24 cases (82.7%) in tumor samples compared with their corresponding normal tissues; CIP2A protein (detected by immunohistochemistry) is found to be over-expressed in 72.2% of 90 lung cancer samples and correlated with poor survival. We test CIP2A expression in 60 patients from Southern China [8], and report that CIP2A is drastically increased in 63.3% (detected by western blotting) or 67.2% (at mRNA level) of tumor specimens compared to adjacent normal tissues (Figure 1).

We show for the first time that CIP2A overexpression is associated with cigarette smoking. In the 60 lung cancer patients examined in this study, 28 of 36 (77.8%) smoker patients show increased expression of CIP2A in the tumor samples compared to their adjacent normal specimens, whereas 10 of 24 (41.7%) nonsmoker cases exhibit up-regulated CIP2A at protein level (p = 0.004) (Table 1). Currently smoking continues to be more prevalent among men (63%) than women (3.8%) [24]. We report here that CIP2A is over-expressed in 30/40 (75%) and 8/20 (40%) in men and women patients (p = 0.008) (Table 1), respectively. We also find that CIP2A is elevated in 17/19 (89.5%) patients with squamous cell carcinoma, whereas 17/35 (48.6%) patients with adenocarcinoma have over-expressed CIP2A (p = 0.003) (Table 1). The multivariate logistic regression analysis clearly demonstrates that smoking is the only significant variable associated with CIP2A overexpression in lung cancer in our setting (p = 0.008) (Table 2). While the overall smoking rate in the North and Northeast sectors is higher than that in the South and East of China, the prevalence of environmental tobacco smoke exposure among nonsmokers in Northern China is significantly higher than that in nonsmokers in Southern China [24], [25]. These may contribute to the difference in CIP2A expression between patients in our setting and Dong's report [23]. Tobacco smoke which causes approximately 5–6 million deaths per year and 31% and 6% of all lung cancer deaths in middle-aged men and women respectively [26], can induce genetic and epigenetic changes and reduce DNA repair capacity [2]. Zhao et al [27] recently showed that in human gastric cell lines, *Helicobacter pylori* infection can induce CIP2A expression via bacterial oncoprotein CagA-caused activation of the Src and MEK/ERK signal pathways. Though NNK (0.1 to 50 µM) fails to upregulate CIP2A in HBEpiC and BEAS-2B cells in up to 18 days in our study (Figure 2), we can not exclude the possibility that NNK may perturb the expression of the oncoprotein in a longer exposure time course, and this hypothesis warrants further investigation.

CIP2A is critical for lung cancer cell growth, since CIP2A knockdown by specific siRNA results in significant inhibition of cell proliferation and transformation in vitro and in vivo (Figure 3 and Figure S1). These data indicate that CIP2A could be an attractive target for novel anti-lung cancer drugs. Interestingly, we identify a natural compound rabdocoetsin B, can down-regulate CIP2A protein in lung cancer cells (Figure 4, A through C) by inhibiting its expression at mRNA level (Figure 4D). Studies show that CIP2A can up-regulate pAkt [15], and our results confirm that CIP2A silencing can decrease pAkt (Figure 5A). We further show that rabdocoetsin B also inhibits pAkt (Figure 5, B through E). Rabdocoetsin B inhibits the growth and induces apoptosis of a variety of lung cancer cells (Figure 6, A through E). Our data thus provide a lead compound for CIP2A-targeting anti-tumor therapeutics.

## Materials and Methods

### Patient samples

Use of the samples was approved by the Institutional Review Board of Institute of Zoology, Chinese Academy of Sciences and The Cancer Hospital, Sun Yat-Sen University. All tumor and adjacent normal tissue samples were obtained with written informed consent from patients at the Cancer Hospital, Sun Yat-Sen University. For RT-PCR analysis, tissue specimens were ground in liquid nitrogen-cooled mortar, RNA was extracted using the Trizol (Invitrogen), and RT-PCR experiments were performed using PrimeScript® One Step RT-PCR Kit Ver.2 (TaKaRa) according to the manufacturer's protocol. The sequences of primers used for *CIP2A* are as follows: forward 5′-CCATATGCTCACTCAGATGATGT-3′,_reverse 5′-GTGTATCATCTCCACAGAGAGTT-3′ [5].

For Western blot assay, tissue specimens were ground in liquid nitrogen-cooled mortar, tissue powder was suspended in lysis buffer (50 mM Tris-HCl (pH 7.4), 150 mM NaCl, 1% triton X-100, 1% sodium deoxycholate, 0.1% SDS, 1 mM Na_3_VO_4_, 1 mM NaF, 1 mM EDTA, 1 mM PMSF, complete protease inhibitor cocktail) and cleared by centrifugation. Equal amounts of samples were separated by SDS-PAGE, transferred to nitrocellulose and immunoblotted with anti-CIP2A or anti-β-actin antibody.

Immunohistochemical assay and scoring of immunoreactivity were performed as described [28]. Formalin-fixed, paraffin-embedded lung cancer tissue specimens (5 µm) were deparaffinized and subjected to a heat-induced epitope retrieval step for 2 min. The H_2_O_2_ (3%) was used to block endogenous peroxidase activity for 10 min. The sections were then washed with PBS. The rabbit polyclonal anti-CIP2A antibody was applied to the slides at a dilution of 1∶500 at 4°C overnight. Detection was achieved with the Immunohistochemistry kit (pv-6001) (Zhongshan Golden Bridge Biotechnology Co., Ltd, Beijing, China) according to the manufacturer's protocol. Sections were colored with 3, 3′-diaminobenzidine (DAB) and counterstained with hematoxylin, dehydrated, treated with xylene, and mounted. CIP2A protein levels were scored as described [29].

### Agents

Rabdocoetsin B was extracted from Rabdosia coetsa by Professor Han-Dong Sun. The 3-(4, 5)-dimethylthiahiazo (-z-y1)-3, 5-di- phenytetrazoliumromide (MTT) was purchased from Amresco, Inc. (Solon, OH). PE Annexin V-7AAD Apoptosis Detection kit was obtained from BD Biosciences (San Jose, CA, USA).

### Antibodies

The antibodies used in this study were as follows: anti-β-Actin (Sigma); anti-casp-9 (C9), anti-casp-8 (1C12); anti-PARP, anti-phospho-p44/42 MAPK, anti-EGFR, anti-Src and anti-β-catenin (Cell signaling), anti-CIP2A (2G10-3B5), anti-pAKT and ATK (Santa Cruz Biotechnology); anti-ERK2 (Abcam); anti-PCNA (Abmart); anti-rabbit or anti-mouse HRP-conjugated secondary antibody (Pierce); Rabbit Polyclonal anti-CIP2A (Novus Biologicals, Inc). Detection was performed by using a Chemiluminescent Western detection kit (Cell Signaling).

### Cell culture

The lung cancer lines NCI-H1975 and A549 and human embryonic kidney HEK-293 cells were obtained from the American Tissue Culture Collection (ATCC) and human embryonic lung fibroblast MRC-5 cells were purchased from the Cell Resource Center, Chinese Academy of Medical Sciences (Beijing). Normal human bronchial epithelial cells (HBEpiC, Catalog Number: 3210) were purchased from ScienCell (ScienCell Research Laboratories, San Diego, California). Human lung squamous carcinoma cell lines L78 and highly metastatic large-cell lung cancer cell lines 95D were obtained from the Cell bank of Chinese Academy of Sciences (Shanghai), and human embryonic lung fibroblast HLF cells were purchased from Kenqiang Instrument Co., Ltd (Shanghai, China). BEAS-2B bronchial epithelial cells were provided by Professor Hongbin Ji at Shanghai Institute for Biological Sciences, Chinese Academy of Sciences. A549, HLF and BEAS-2B cells were cultured in Dulbecco modified Eagle medium (DMEM) containing 10% fetal bovine serum (FBS; Gibco/BRL, Grand Island, NY), 100 U/ml penicillin, 100 µg/ml streptomycin. HBEpiC cells were cultured in a serum-free Bronchial Epithelial Cell Medium (BECM, Cat. No. 3211, ScienCell Research Laboratories) containing essential and non-essential amino acids, vitamins, hormones, growth factors and trace minerals. L78, 95D and NCI-H1975 cells were cultured in RPMI 1640 supplemented with 10% fetal bovine serum (FBS; Gibco/BRL, Grand Island, NY), 100 U/ml penicillin, 100 µg/ml streptomycin. MRC-5 cells were cultured in MEM/EBSS medium supplemented with non-essential amino acids, 10% fetal bovine serum (FBS; Gibco/BRL, Grand Island, NY), 100 U/ml penicillin and 100 µg/ml streptomycin.

### Assessment of cell proliferation and apoptosis

Cells were treated with Rabdocoetsin B for indicated concentration and time points. Cell proliferation was determined using MTT assay. Cell viability was estimated by trypan blue dye exclusion. Externalization of phosphatidylserine was tested using a PE Annexin V-7 AAD Apoptosis Detection kit (BD Biosciences, San Jose, CA) according to manufacturer's instruction.

### Quantitative real-time PCR

Quantitative real-time PCR was performed in CFX^TM^96 Real Time System (Bio-Rad) using SYBR® Premix Ex Taq™(Perfect Real Time) (TaKaRa Code: DRR041) according to the manufacturer's instruction. Primers for CIP2A and Actin were as follows: CIP2A: forward sequence, 5′-TGCGGCACTTGGAGGTAATTTC-3′, reverse sequence, 5′-AGCTCTACAAGGCAACTCAAGC-3′; Actin: forward sequence, 5′-ATCGTCCACCGCAAATGCTTCTA-3′, reverse sequence, 5′-AGCCATGCCAATCTCATCTTGTT-3′. Levels of CIP2A mRNA were expressed as the ratio versus actin based on the CT values.

### siRNA assays

Using HiPerFect Transfection Reagent (Qiagen, Crawley, UK) according to the manufacturer's protocol, cells were transfected with 100 nM double-stranded siRNA oligonucleotides [3]. The siRNA sequences were 5′-CUGUGGUUGUGUUUGCACUTT-3′ (CIP2A siRNA1), 5′-ACCAUUGAUAUCCUUAGAATT-3′ (CIP2A siRNA2) [3], and 5′-UUCUCCGAACGUGUCACGUTT-3′ (negative control (NC) siRNA).

### Clonogenic assay

For foci formation, A549 or L78 cells transfected with negative control or CIP2A-specific siRNA were seeded in triplicate onto 35 mm plates (300 cells per plate). After 14 days of culturing, cells were stained with Giemsa and clones containing more than 50 cells were counted. For soft-agar colony formation assay, cells were suspended in 1 ml DMEM (for A549 cells) or RPMI 1640 (for L78 cells) containing 0.3% low-melting-point agarose (Amresco, Solon, OH) and 10% FBS and plated on a bottom layer containing 0.6% agarose in 35 mm plat (1,000 cells/plate) in triplicate. After 2-3 weeks culturing, plates were stained with Giemsa and colonies were counted using a light microscope.

### Murine models

All animal studies were conducted according to protocols approved by the Animal Ethics Committee of the Institute of Zoology, Chinese Academy of Sciences, with the approval ID of AEC2010070202. All mice used in this study were bred and maintained in a specific pathogen-free environment. Nude mice (n = 8) were injected subcutaneously with A549 cells (4×10^6^) transfected with NC or CIP2A-specific siRNA into right and left flanks respectively, and tumor was calculated as described [12].

### Statistical analysis

Differences between data groups were evaluated for significance using Student *t*-test of unpaired data, χ^2^ test or one-way analysis of variance and Bonferroni post-test. The tumor volume was analyzed with one-way ANOVA and independent sample *t* test using the software SPSS 12.0 for Windows (Chicago, IL). The association between CIP2A high expression and clinicopathological feature was assessed using either χ^2^ test or the Fisher exact test, and a backward stepwise multivariate logistic regression analysis was carried out to investigate the most significant variables related to CIP2A over-expression after adjustment. P values <.05 were considered statistically significant. All experiments were repeated at least three times and the data were presented as the mean±SD unless noted otherwise.

## Supporting Information

Figure S1 **The effects of CIP2A depletion on the L78 cells' growth and trasnsformation.** (A): Western blot analysis of CIP2A protein expression in L78 cells 72 h after transfection with NC or CIP2A-specific siRNA. (B and C): Flat plate clone formation assay for clonogenic activity of L78 cells 72 h after transfection with NC or CIP2A-specific siRNA. (B): Representative light microscopy images. (C): Quantitation of foci counting. Shown is mean+SD of three independent experiments. (D and E): Soft-agar colony formation assay of L78 cells transfected with NC or CIP2A-specific siRNA. (D): Representative light microscopy images. (E): Quantitation of foci counting.
(TIF)
