# Supplementary figures and images for: Overexpression and Small Molecule-Triggered Downregulation of CIP2A in Lung Cancer
Source: PLoS One. 2011 May 31;6(5):e20159. doi: 10.1371/journal.pone.0020159 (PMC3105001; doi:10.1371/journal.pone.0020159)

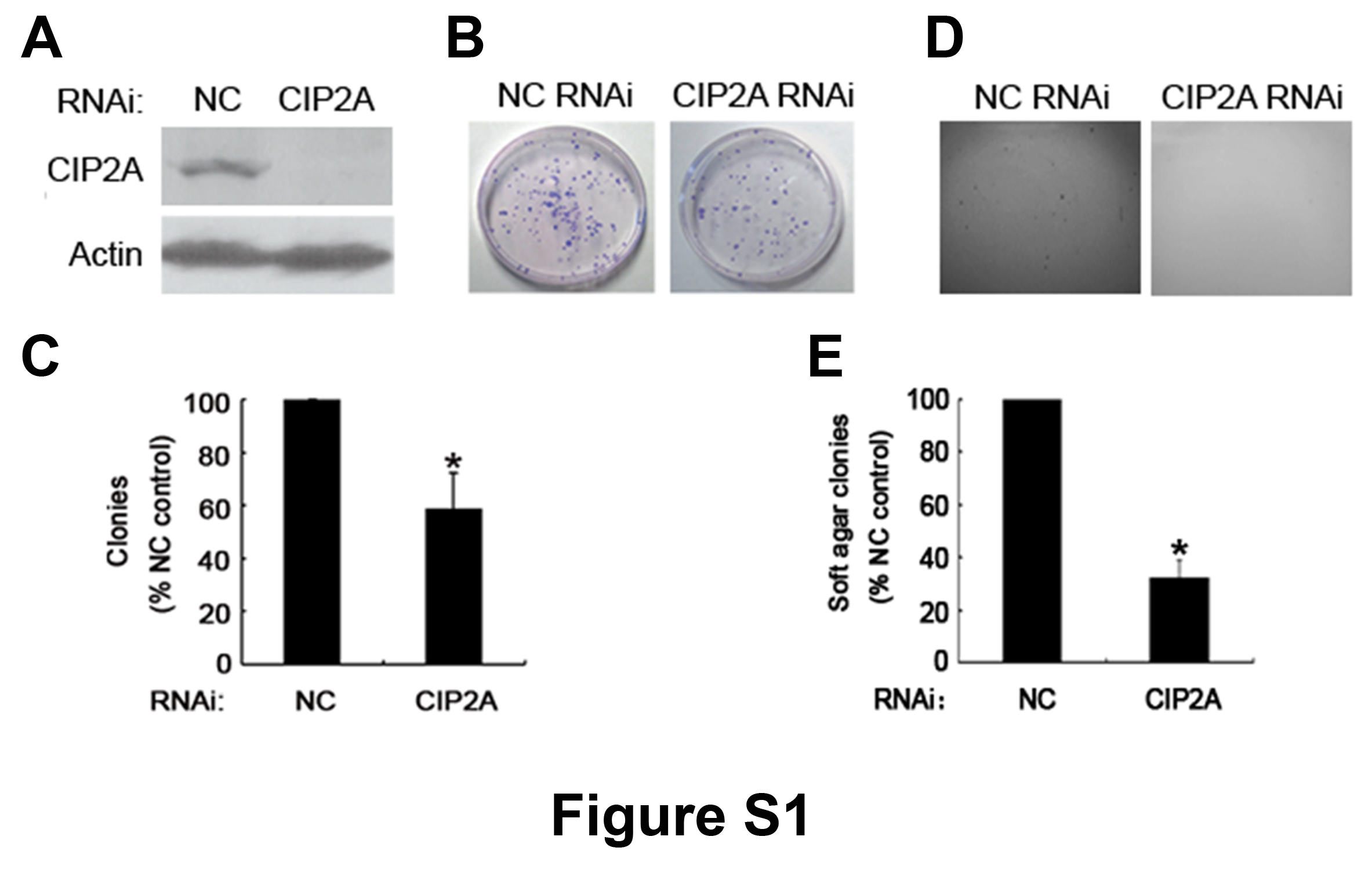

Supplement: Figure S1 — The effects of CIP2A depletion on the L78 cells' growth and trasnsformation. (A): Western blot analysis of CIP2A protein expression in L78 cells 72 h after transfection with NC or CIP2A-specific siRNA. (B and C): Flat plate clone formation assay for clonogenic activity of L78 cells 72 h after transfection with NC or CIP2A-specific siRNA. (B): Representative light microscopy images. (C): Quantitation of foci counting. Shown is mean+SD of three independent experiments. (D and E): Soft-agar colony formation assay of L78 cells transfected with NC or CIP2A-specific siRNA. (D): Representative light microscopy images. (E): Quantitation of foci counting. (TIF) [file pone.0020159.s001.tif]
